# Screening, Identification and Efficacy Evaluation of Antagonistic Bacteria for Biocontrol of Soft Rot Disease Caused by *Dickeya zeae*

**DOI:** 10.3390/microorganisms8050697

**Published:** 2020-05-09

**Authors:** Jieling Li, Ming Hu, Yang Xue, Xia Chen, Guangtao Lu, Lianhui Zhang, Jianuan Zhou

**Affiliations:** 1 Guangdong Laboratory for Lingnan Modern Agriculture, Guangdong Province Key Laboratory of Microbial Signals and Disease Control, Integrative Microbiology Research Centre, South China Agricultural University, Guangzhou 510642, China; Jielingbangbang@126.com (J.L.); hm13@stu.scau.edu.cn (M.H.); 18819455948@163.com (Y.X.); chenx1257@163.com (X.C.); lhzhang01@scau.edu.cn (L.Z.); 2 State Key Laboratory for Conservation and Utilization of Subtropical Agro-bioresources, College of Life Science and Technology, Guangxi University, Nanning 530004, China; lugt@gxu.edu.cn

**Keywords:** antagonistic bacterial screening, species identification, determination of biocontrol effect

## Abstract

*Dickeya zeae* is the causal agent of bacterial soft rot disease, with a wide range of hosts all over the world. At present, chemical agents, especially agricultural antibiotics, are commonly used in the prevention and control of bacterial soft rot, causing the emergence of resistant pathogens and therefore increasing the difficulty of disease prevention and control. This study aims to provide a safer and more effective biocontrol method for soft rot disease caused by *D. zeae*. The spot-on-lawn assay was used to screen antagonistic bacteria, and three strains including SC3, SC11 and 3-10 revealed strong antagonistic effects and were identified as *Pseudomonas fluorescens*, *P. parafulva* and *Bacillus velezensis*, respectively, using multi-locus sequence analysis (MLSA) based on the sequences of 16S rRNA and other housekeeping genes. *In vitro* antimicrobial activity showed that two *Pseudomonas* strains SC3 and SC11 were only antagonistic to some pathogenic bacteria, while strain 3-10 had broad-spectrum antimicrobial activity on both pathogenic bacteria and fungi. Evaluation of control efficacy in greenhouse trials showed that they all restrained the occurrence and development of soft rot disease caused by *D. zeae* MS2 or EC1. Among them, strain SC3 had the most impressive biocontrol efficacy on alleviating the soft rot symptoms on both monocotyledonous and dicotyledonous hosts, and strain 3-10 additionally reduced the occurrence of banana wilt disease caused by *Fusarium oxysporum* f. sp. *cubensis*. This is the first report of *P. fluorescens*, *P. parafulva* and *B. velezensis* as potential bio-reagents on controlling soft rot disease caused by *D. zeae*.

## 1. Introduction

*Dickeya zeae*, formerly named *Erwinia chrysanthemi* pv. *zeae*, is the causal agent responsible for maize stalk rot, rice foot rot and banana soft rot diseases in different parts of the world [1,2,3,4,5]. It has been found in Asia, America, Australia, Europe and Africa, threatening agricultural production [6]. It can infect at least 24 types of dicotyledons and 22 types of monocotyledons [6], although it is mostly isolated from monocots in Southeast Asian countries in nature [6], while other *Dickeya* species mainly infect dicotyledons [7,8,9].

In recent years, *D. zeae* has caused huge economic losses to the maize, rice and banana industries [6], and meanwhile has expanded its host range [6]. Maize stalk rot has been reported in USA, Brazil, France, Italy, Senegal, Cuba, Egypt, Mexico, India, Korea, Iran, Japan, China and Thailand [6]. Rice foot rot mainly occurs in south China, with yield loss ranging from 10% to 30%, and even over 60% in some rice planting regions in China [10,11,12,13,14]. It also threatened different rice-cultivated areas in many Southeast Asian countries and Europe [6,15]. On banana, the incidence and severity of soft rot disease has increased since it first broke out in Guangzhou in 2009 [16], resulting in a 20% to 70% incidence, and even 90% in some plantations from 2010 to 2012 in Guangdong Province [4]. Banana infected by *D. zeae* was also found in Ivory Coast, Jamaica, Panama and Martinique [1,6]. The natural host range of *D. zeae* has been extended to hyacinth and clivia [6,17]. The pathogen is able to spread via water, survive on field weeds and plant debris, increasing the difficulty of prevention and control of soft rot disease [1].

At present, chemical bactericides and resistant cultivars are usually applied to control plant bacterial diseases. However, large scale application of toxic chemicals may cause environmental hazards, the development of resistance in pathogen populations and damage to non-target organisms [18]. Furthermore, the long-term and large-scale cultivation of single resistant cultivar may result in adaptive variation of *D.* zeae, creating favorable conditions for the proliferation and accumulation of virulent strains and eventually causing the loss of cultivar resistance. Comparatively speaking, biocontrol is a powerful alternative to the use of synthetic chemicals, and an environmentally friendly means to control plant diseases [19]. The main biocontrol strategies against soft rot bacteria comprise the use of bacterial antagonists, natural predators (including bacteriophages and bacteria), quorum-quenching bacteria and induction of plant systemic resistance [20,21]. Previous studies have shown that some bacteria with antagonistic activities against *Dickeya* spp. have good efficacy in laboratory conditions. For instance, potato tubers treated with *Serratia plymuthica* A30 can relieve the severity of potato soft rot caused by *D. solani* during storage and reduce the transmission of pathogenic bacteria from mother tubers to progeny tubers during field cultivation [22]. Recent reports suggest that a combination of biocontrol strains could be a potential strategy to limit the potato soft rot and blackleg diseases [23,24]. In addition, a *Bacillus amyloliquefaciens* strain D2WM was found to have good inhibitory activity on *D. chrysanthemi*, and macrolactin A was identified as the key antibacterial substance [25]. As for the diseases caused by *D. zeae*, *Rahnella aquatilis* and *Erwinia persicinus* were reported to significantly reduce the severity of tissue maceration on hyacinth bulbs [17], and *B. subtilis* strain A2 was also found to have bacteriostatic effect against soft rot of *Guzmania denise* [26].

In view of the severity of rice and banana diseases caused by *D. zeae* in China and the lack of bioreagents for controlling the bacterial soft rot disease in fields nowadays, we screened antagonistic bacteria from different environmental samples to explore an effective and durable method for soft rot disease control. The findings in this study provide *Pseudomonas fluorescens* SC3, *P. parafulva* SC11 and *Bacillus velezensis* 3–10 as *Dickeya* novel biocontrol agents for potential application on bacterial disease control.

## 2. Materials and Methods

### 2.1. Isolation of Bacterial Strains

From 2017 to 2019, environmental samples including rhizospheric soils, asymptomatic plant (cabbage, ginger and banana in vegetative growth period) tissues and soft rot rice tissues in the tillering stage were collected from Guangdong, Guangxi and Sichuan Provinces. Bacteria were isolated with methods previously described with minor modifications [27]. Briefly, 4 g of plant tissues or soil samples was added in 36 mL of sterile water and shaken for 20 min. Then, 100 µL of the suspension was transferred for serial dilution up to 10^−6^, and plated on lysogeny broth (LB, per liter contains 10.0 g of tryptone, 5.0 g of yeast extract and 10.0 g of NaCl, pH = 7.0) agar (1.5% *w*/*v*) plates at 28 °C for 16–18 h. Single colonies were grown overnight in 1 mL of LB medium with shaking at 200 rpm at 28 °C.

### 2.2. Screening of Antagonistic Bacteria

The antagonistic activities against *D. zeae* of the above isolated bacteria were tested by spot-on-lawn assay as previously described [27]. *D. zeae* rice strain EC1 [28], banana strains MS2 and MS3 [6] and antagonistic candidate strains were grown overnight at 28 °C in 10 mL of liquid LB medium with shaking at 200 rpm, and adjusted to the same cell density (OD_600_ = 1.5). Specifically, 200 μL of *D. zeae* overnight culture was added into 20 mL of 1% agarose (cooled to 50 °C), mixed and poured onto the surface of a LB agar plate (13 cm × 13 cm), dried at room temperature and then punched with a 5 mm puncher. Finally, 20 μL of overnight antagonistic candidate culture was added into the hole. An equal volume of LB medium was used as the blank control. Plates were incubated at 28 °C for 24 h. Antagonistic activity was evaluated based on the size of the growth inhibition zone. The selected antagonistic bacteria were tested for three times.

The antibacterial activities of *D. zeae* strains against the isolated antagonistic bacteria were also determined using the above methods.

### 2.3. Identification of Antagonistic Bacteria

To identify the screened antagonistic bacteria, genomic DNA was extracted using MasterPure DNA purification kit (EPICENTRE Biotechnologies, Madison, WI, USA), and sequences of 16S rRNA gene were amplified using primers 27F and 1492R [29] and cloned into the pUC19-T vector (New England Biolabs, Beijing, China) for sequencing. The obtained sequences were submitted to NCBI (National Center for Biotechnology Information) for sequence alignment. To clarify the taxonomic status of the antagonistic bacteria, multilocus sequences of the strains were analyzed, including partial sequences of DNA gyrase subunit B (*gyrB*), RNA polymerase β subunit (*rpoB*) and RNA polymerase subunit D (*rpoD*) genes for strains SC3 and SC11, using primer pairs of GyrBPUN1F/GyrBPUN1R, LAPS5F/LAPS27R and PsEG30F/PsEG790R, respectively [30,31,32,33], and DNA gyrase subunit A (*gyrA*) and *gyrB* for strain 3-10 using primer pairs of GYRA F/GYRA R and GYRB UP1/GYRB UP2r, respectively, with conditions described in corresponding references [34,35]. All the resultant sequences were aligned and verified before submitting to the GenBank database with accession nos. as: MN511732 (16S rRNA), MN648418 (*gyrB*), MN648413 (*rpoB*) and MN648414 (*rpoD*) for strain SC3; MN511735 (16S rRNA), MN648415 (*gyrB*), MN648411 (*rpoB*) and MN648412 (*rpoD*) for strain SC11; MN515140 (16S rRNA), MN648416 (*gyrA*) and MN648417 (*gyrB*) for strain 3-10.

### 2.4. Phylogenetic Analysis of Antagonistic Bacteria

To reveal the evolutionary relationships of the target antagonistic strains SC3, SC11 and 3-10, phylogenetic analysis was performed by constructing a joint phylogenetic tree for each strain. Firstly, sequences of each gene of related strains were obtained from GenBank database and aligned with ClustalW software in the MEGA6.0 software package (Pennsylvania State University, University Park, PA, USA) to cut the sequences in a same size as 16S rRNA =1498 bp, *gyrB* = 749 bp, *rpoB* = 1230 bp and *rpoD* = 740 bp for constructing trees of strains SC3 and SC11, and 16S rRNA = 1511 bp, *gyrA* = 1025 bp and *gyrB* = 1248 bp for constructing a tree of strain 3–10. Secondly, the trimmed sequences of every strain were concatenated in the same order and re-aligned using ClustalW. Finally, joint phylogenetic trees were built using the neighbor-joining method with maximum composite likelihood model in 1000 bootstrap replicates.

### 2.5. Determination of Antimicrobial Spectrum of Strains

Pathogenic microorganisms to be tested in this study are listed in Table 1; of these, bacterial pathogens were grown in LB medium except *Ralstonia solanacearum* EP1 in TTC (2,3,5-Triphenyte-trazoliumchloride) medium (per liter contains 10.0 g of tryptone, 4.0 g of glucose, 1.0 g of yeast extract, 1.0 g of casein acid hydrolysates and 0.5 mg 2,3,5-Triphenyte-trazoliumchloride) until OD_600_ = 1.5, and fungi were grown on potato-dextrose-agar (PDA) (TOPBIO, Zhaoyuan, China) plates at 28 °C until hypha grew to the plate edge. For testing the antimicrobial activities of the antagonistic bacteria against bacterial pathogens, the spot-on-lawn assay was also used as above. All the tested pathogenic bacterial cultures (200 μL) listed in Table 1 were added into 20 mL of 1% agarose (cooled to 50 °C) and poured onto the surface of LB agar plates except *R. solanacearum* EP1 poured onto the surface of TTC plate. LB medium was used as the blank control. All the plates were incubated at 28 °C for 3 days and the radius sizes of the growth inhibition halos were measured. For testing the inhibitory activities of the antagonistic bacteria against pathogenic fungi, a plate confrontation assay was performed using the method previously described [6], except for the bioassay of inhibitory activities against the mating of *Sporisorium scitamineum*, which was referred to the method described by Liu et al. [36]. LB medium was used in the same way as a negative control. All the plates were incubated at 28 °C until hypha in the negative control grew to reach the edges of the plates or slices. The distance between the antagonistic bacteria and the fungal hyphal edge was measured. The experiment was repeated three times.

### 2.6. Determination of Greenhouse Control Effect of Antagonistic Bacteria

Firstly, pathogenic bacteria *D. zeae* strains EC1 and MS2 and three antagonistic bacterial strains were respectively cultured in 10 mL of LB medium at 28 °C, shook at 200 rpm for about 10 h (OD_600_ ≈ 1.8), and mixed at a 1:1 (*D. zeae*: antagonistic bacterium) ratio. Secondly, an aliquot of the mixture was inoculated to the monocotyledonous and dicotyledonous hosts. Inoculation on dicotyledonous plants was performed as in a previous study [37] with minor modifications. Briefly, carrot (cv. Changhong), potato tuber (cv. Luyin No. 1) and Chinese cabbage (cv. Tianjinlv) were surface-disinfected by 70% ethanol, cut into slices and dried for about 20 min at room temperature. An aliquot of 1 µL mixture was inoculated on the center of the tissue slices. After inoculation, tissue slices were placed on wet sterilized filter paper in 12 cm-petri dishes and kept in a growth chamber with conditions of 28 ± 2 °C and 75 ± 15% relative humidity for about 18 h. The area of lesions was measured using Image J 1.52a (The National Institutes of Health, Bethesda, MD, US). Tissue slices inoculated with LB medium, MS2 and pure antagonistic bacteria were respectively served as blank, positive and negative controls.

For monocotyledons, rice seeds (cv. CO39) were immersed in 70% ethanol for 1 min, rinsed three times with distilled water, germinated in petri dishes and then grown (20 seeds) in plastic pots filled with sterile soils up to 5 cm below their mouth. After three weeks of germination, an aliquot of 10 mL mixture was irrigated into the pots near stem bases with needle punctures. Inoculation on banana was referred to in our previous study [43]. Specifically, tissue culture banana seedlings (*Musa sapientum* ABB cv. Guangfen No. 1) were grown in greenhouse (28 ± 2 °C with 75% ± 15% relative humidity, 12 h alternating light and dark cycles) until the four- or five-leaf stage. An aliquot of 200 µL mixture was injected into the pseudostems of banana seedlings (10 seedlings). Plants were incubated at 28 ± 2 °C with 75 ± 15% relative humidity and 12 h alternating light and dark cycles for 7 days. Distilled water was added when necessary and disease was assessed using the virulence scoring previously described [43,44]. Seedlings inoculated with LB medium and pure *D. zeae* were respectively served as blank and positive controls.

Based on the effective inhibitory activity of strain 3-10 on fungal pathogen, we tested its efficacy on controlling banana wilt disease caused by *F*. *oxysporum* f. sp. *cubensis* FOC4, since we found that banana wilt disease and soft rot disease often occurred at the same time in the field according to our investigations. Strain 3-10 was cultured in LB medium overnight and resuspended with double distilled water (ddH_2_O) to a final concentration of OD_600_ = 0.1. FOC4 strain was cultured on PDA plates for about 5 days until the hypha reached the plate edge. Plates were washed with ddH_2_O to collect hypha and conidia. Half roots of each banana seedling (30 seedlings) were randomly cut off, among which 20 were soaked in pure FOC4 suspension and 10 were soaked in ddH_2_O for 20 min, and re-planted in sterilized pots, respectively. Then 100 mL of 3-10 cell suspension and ddH_2_O was respectively drenched into the soils of each 10 FOC4-soaked seedlings, while 100 mL of ddH_2_O was also drenched into the soils of the ddH_2_O-soaked seedlings. Wilt disease score was assessed as described above.

Three independent trials were carried out in triplicate for all the inoculation work.

### 2.7. Testing of Antibiosis Activities of the Antagonistic Bacteria in Liquid and Solid Media

To determine the inhibitory activities of the extracellular metabolites from the three antagonistic bacteria, supernatants were obtained by filtrating the overnight bacterial cultures using 0.22-μm filters, and 50 μL of which were added into the wells of the MS2 lawn plates. In addition, to compare the inhibitory activities between the supernatants and bacteria, 0.5 μL of the OD_600_ = 1.5 bacterial cultures were spotted onto the MS2 lawn plates. The plates were incubated at 28 °C for 2 days.

To test the inhibitory activities of the three antagonistic strains in solid culture, we streaked lines of each overnight antagonistic bacterial culture on LB agarose plates (10 cm × 10 cm) and incubated the plates at 28 °C for 36 h. After incubation, the agar strips with bacterial lawns were cut off and 1 μL of MS2 culture (OD_600_ = 1.5) serial dilutions (10^0^ to 10^−5^ dilutions) were progressively spotted onto the remaining, cell-free agar strips. Some of the cell-free agar strips were melted using a microwave oven, and MS2 dilutions were spotted in the same way onto the melted agar plates in order to test the thermal sensitivity of the secreted metabolites from the antagonistic strains. All the plates were incubated at 28 °C for 18 h.

To test the inhibitory activity of strain SC3 in liquid culture, cell-free supernatants were added into 5-fold-concentrated LB agarose medium (cooled to about 60 °C) in a ratio of 7:3, and 1 μL of MS2 culture (OD_600_ = 1.5) dilutions were progressively spotted onto the plates. Supernatants were treated with trypsin in a final concentration of 2 mg/mL for 30 min to determine the property of the antagonistic substances from strain SC3. The plates were incubated at 28 °C for 2 days. LB agarose plates were used as the negative control. The experiment was repeated three times.

## 3. Results

### 3.1. Isolation of Strains with Antagonistic Activities

**Figure 1:**
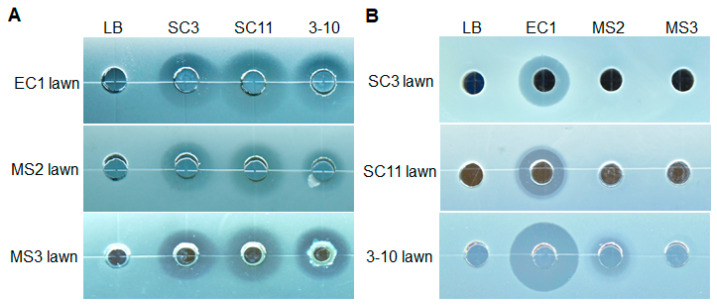
Inhibitory activities of antagonistic strains and *Dickeya zeae* pathogens in spot-on-lawn assay. The plates were incubated at 28 °C for 24 h. (**A**) the antagonistic activities of strains SC3, SC11 and 3-10 against pathogenic *D. zeae* strains EC1, MS2 and MS3; (**B**) inhibitory activities of *D. zeae* pathogens against antagonistic strains SC3, SC11 and 3-10.

Environmental samples were collected from Guangdong, Guangxi and Sichuan Provinces. A total of 3592 bacteria were isolated and tested on the antagonistic activities against *D. zeae* strains, among which 55 strains showed inhibitory activities. Three strains, designated as SC3, SC11 (both isolated from stem bases of diseased rice plants in Sichuan) and 3-10 (isolated from ginger rhizosphere in Guangdong), exhibited a strong inhibitory effect on *D. zeae* EC1, MS2 and MS3 strains on plates in spot-on-lawn assay, with strains SC3 and SC11 forming a larger transparent growth inhibition zone than strain 3-10 in the same cell density (Figure 1A).

### 3.2. Susceptibility of three Antagonistic Bacteria to Metabolites Secreted by D. zeae Strains

Theoretically, a good biocontrol strain suitable for field application should not be inhibited by the pathogenic target. In this study, we wondered whether and how much the metabolites produced by strains MS2 and EC1 could inhibit the growth of the three antagonistic bacteria, since we previously found that both of them could produce antibiotic-like metabolites, while strain MS3 could not [6,28]. From the result in Figure 1B, *D. zeae* rice strain EC1 showed obvious inhibitory activity against all three antagonistic bacteria (Gram-positive and Gram-negative bacteria), whereas *D. zeae* banana strain MS2 only had a little inhibitory activity against strain 3-10 (Gram-positive bacterium) and strain MS3 had none.

### 3.3. Identification of Antagonistic Strains SC3, SC11 and 3-10

**Figure 2:**
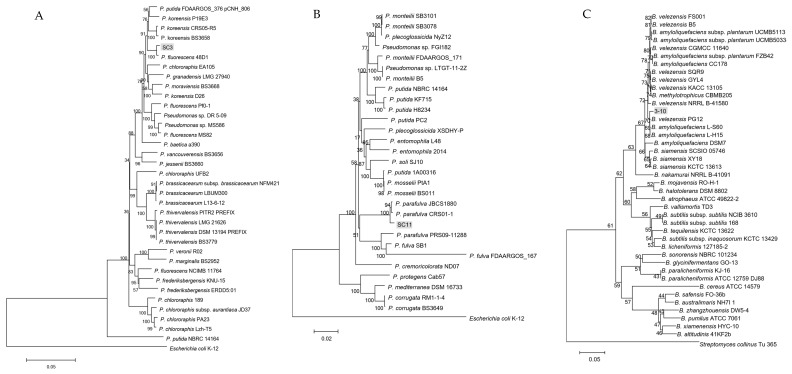
Joint phylogenetic trees based on the concatenated nucleotide sequences of the 16S rRNA, *gyrB*, *rpoB* and *rpoD* genes of strains SC3 (**A**), SC11 (**B**), and the 16S rRNA, *gyrA* and gyr*B* genes of strain 3-10 (**C**). Consensus sequences of every gene from related strains were aligned with ClustalW and trimmed in the same sizes. All the sequences from the same strain were assembled to construct a joint neighbor-joining tree. Bootstrap values after 1000 replicates are expressed as percentages. Scale bar denotes nucleotide substitutions per site.

Alignment of 16S rRNA sequences of the three strains in GenBank database revealed that strains SC3 and SC11 belonged to the *Pseudomonas* genus, while 3-10 belonged to *Bacillus*. To identify the specific taxonomic status of the three antagonistic strains, MLSA analysis was performed based on the joint sequences of 16S rRNA, *gyrB*, *rpoB* and *rpoD* for strains SC3 and SC11, and 16S rRNA, *gyrA* and *gyrB* for strain 3-10. Phylogenetic trees of concatenated sequences indicated that antagonistic strains SC3, SC11 and 3-10 belonged to *Pseudomonas fluorescens*, *P. parafulva* and *Bacillus velezensis*, respectively (Figure 2).

The 16S rRNA sequence of strain SC3 was 99.0% identical to *P. fluorescens* strains 48D1 and Pfo-1. The alignment of the other three housekeeping gene sequences (*gyrB*, *rpoB* and *rpoD*) showed that SC3 was most similar to *P. fluorescens* 48D1 and LMG14673. The joint phylogenetic tree indicated that SC3 was clustered together with *P. fluorescens* 48D1 (Figure 2A). Thus, these data established that strain SC3 belonged to species *P. fluorescens*.

The 16S rRNA sequence of strain SC11 was 99.8% identical to *P. parafulva* JBCS1880 and CRS01-1, and 99.6% identical to *P. fulva* CI-11, IN78 and Z67zhy. The alignment of the other three gene sequences revealed over 98% identity to *P. parafulva* JBCS1880 and CRS01-1. The joint phylogenetic tree indicated that SC11 was closest to *P. parafulva* JBCS1880 and CRS01-1 (Figure 2B). Therefore, we assigned strain SC11 as species *P. parafulva*.

The 16S rRNA sequence of strain 3-10 was 99.6% homologous to *B. velezensis* GH1-13, GYL4 and *B. siamensis* IHBB14741, 99.73% identical to *B. subtilis* P52, and 99.3% identical to some *B. amyloliquefaciens* strains. The alignment of the *gyrA* and *gyrB* sequences showed that it was over 98% identical to some strains of *B. velezensis*, *B. amyloliquefaciens* and *B. methylotrophicus*. The joint phylogenetic tree revealed that strain 3-10 was most relative to *B. velezensis* PG12 (Figure 2C). In summary, we clarified strain 3-10 as species *B. velezensis*.

### 3.4. Antimicrobial Spectrum of Antagonistic Bacteria SC3, SC11 and 3-10

In order to know the differences in the toxic metabolites from these three antagonistic strains, we measured their inhibitory activities against some important pathogenic microorganisms. The results show that strains SC3 and SC11 only affected the growth of bacterial pathogens including *D. zeae* strains EC1, MS2 and MS3, *D. fangzhongdai* HK1, *D. dadantii* 3937, *Ralstonia solanacearum* EP1 and *Xanthomonas campetris* pv. *campetris* Xcc1 (Table 1), whereas strain 3-10 inhibited both all the above bacterial pathogens and the tested fungal pathogens including *Colletotrichum capsica*, *C. gloeosporioides*, *Fusarium oxysporum* f. sp. *cubensis* FOC4, *Rhizoctonia solani* AG-1 IA, *Magnaporthe oryzae* B157 and *Sporisorium scitamineum* with different inhibition rates (Table 1). SC3 and SC11 are both *Pseudomonas* strains, and they showed a similar antimicrobial spectrum in this study, except that strain SC11 had weak and strong inhibitory activities against the growth of *Pantoea* strains and *B. velezensis* 3-10, respectively, while SC3 had no and weak antibiosis against *Pantoea* strains and *B. velezensis* 3-10, respectively (Table 1, Figure S1). They both had strong inhibition against the growth of *Dickeya* bacteria and no effect on fungal pathogens (Table 1). The *Bacillus* strain 3-10 had the widest antimicrobial spectrum, and the strongest antimicrobial effect on all the tested strains except on the *Dickeya* bacteria (Table 1). It can also inhibit the sexual mating (to form dikaryons) of *S. scitamineum* by restraining the growth of fungal haploid cells (Table 1 and Figure S2). 

### 3.5. Greenhouse Control Effect of Antagonistic Bacteria SC3, SC11 and 3-10

**Figure 3:**
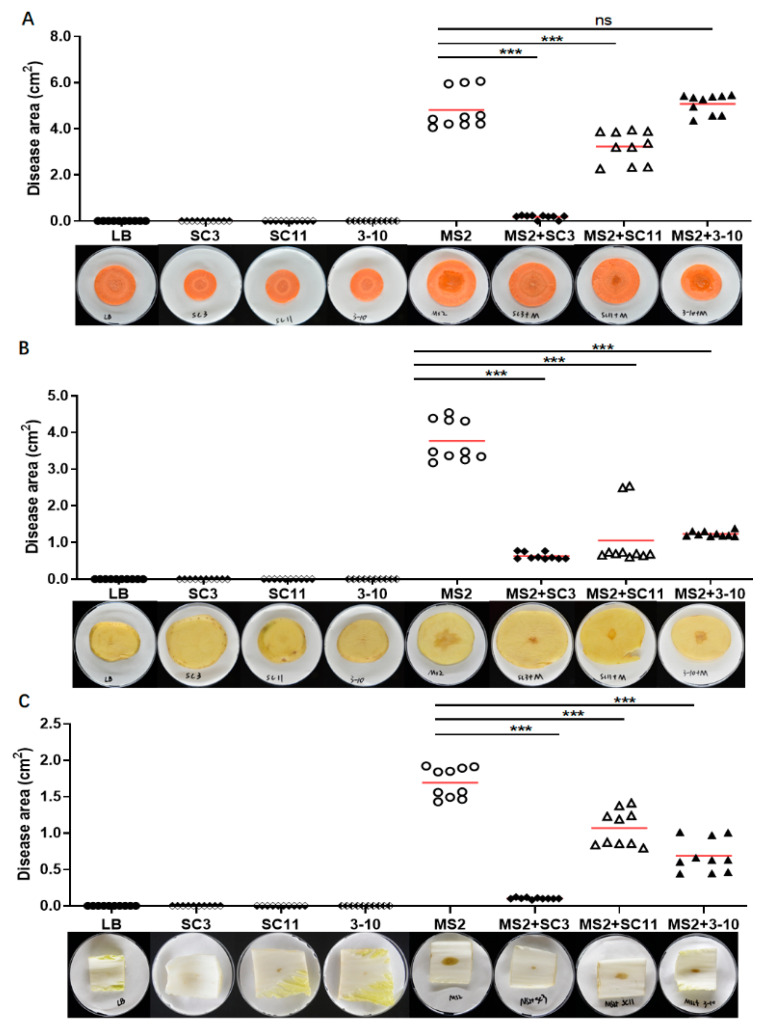
Biocontrol efficacy of antagonistic bacterial strains against soft rot disease on dicotyledonous carrot (**A**), potato (**B**) and Chinese cabbage (**C**). Both MS2 and antagonistic bacteria were grown in lysogeny broth (LB) medium till OD_600_ ≈ 1.8. One microliter of LB, LB+antagonistic bacteria, LB+MS2 and MS2+antagonistic bacterium was respectively spotted onto the center of tissue slices. The rotting area was measured by Image J 1.52a, and the data were subjected to unpaired two-tailed t-test analysis by Graphpad Prism 8.4.1 (GraphPad Software, San Diego, CA, USA) (ns: no statistical significance, *** *p* < 0.001). The marks in the plot area represent the diseased area on 10 inoculated plant slices, and the red lines represent the average values of diseased area.

**Figure 4:**
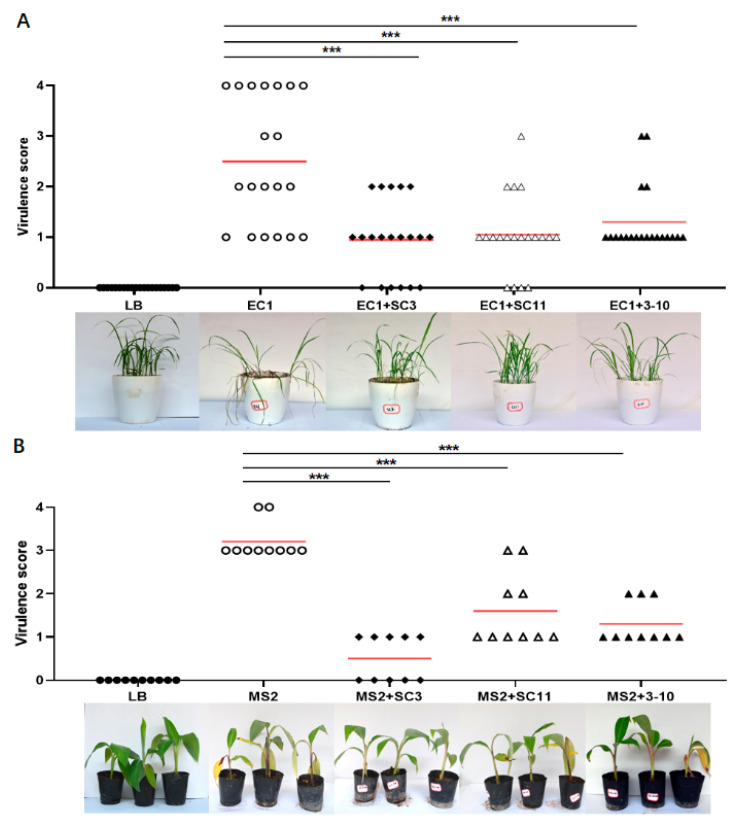
Biocontrol efficacy of antagonistic bacterial strains against rice foot rot and soft rot diseases on monocotyledonous rice (**A**) and banana seedlings (**B**). EC1, MS2 and antagonistic bacteria were grown in LB medium till OD_600_ ≈ 1.8. Ten milliliters of LB, EC1+LB and EC1+antagonistic bacterium was respectively irrigated into the pots with 20 rice seedlings after needle punctures on stem bases. Two hundred microliter of LB, MS2+LB and MS2+antagonistic bacteria was respectively injected into the pseudostems of banana seedlings. Plants were incubated at 28°C with 12 h alternating light-dark cycles for 7 days, and disease was assessed by using virulence scoring method described previously. Data were subjected to unpaired two-tailed t-test analysis by Graphpad Prism 8.4.1 (*** *p* < 0.001). The marks in the plot area represent the virulence scores of the inoculated plant seedlings, and the red lines represent the average virulence scores.

In this study, we measured the biocontrol potential of the above three identified strains against soft rot disease on carrot, potato, Chinese cabbage, rice and banana seedlings under greenhouse conditions. The results show that the addition of each of the three antagonistic strains obviously relieved the symptoms of soft rot on both dicotyledonous (Figure 3) and monocotyledonous hosts (Figure 4) except strain 3-10 on carrot, which showed no statistical significance difference from the MS2 positive control (Figure 3A). Among the three strains, SC3 showed the best biocontrol effect on all of the tested hosts (Figure 3 and Figure 4). By comparison with the plant tissues inoculated with *D. zeae* MS2, which extensively decayed within 24 h, the rotting lesions on tissue discs inoculated with MS2 and SC3 mixture spread very slowly (Figure 3), even after three days of inoculation (data not shown). Additionally, the rice and banana seedlings inoculated with MS2 and SC3 mixture successfully survived and thrived, even after two weeks (data not shown), whereas they almost damped off in 7 days in the positive control (Figure 4).

**Figure 5:**
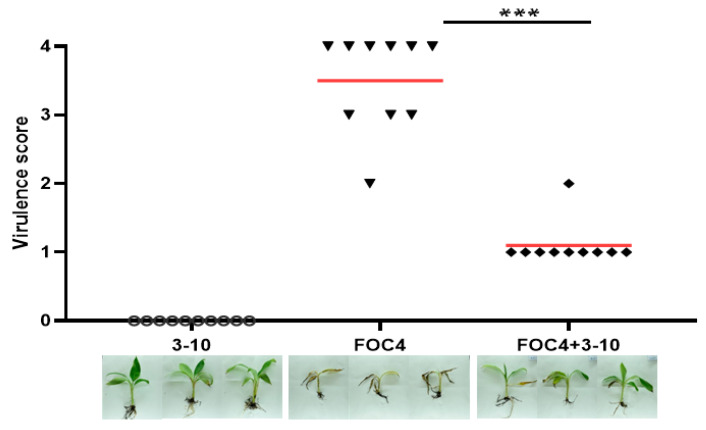
Biocontrol efficacy of strain 3-10 against banana wilt caused by *Fusarium oxysporum* f. sp. *cubensis* FOC4. Strain 3-10 was cultured in LB medium overnight and resuspended with ddH_2_O to a final concentration of OD_600_ = 0.1. FOC4 strain was cultured on PDA plates for about 5 days and washed with ddH_2_O to collect hypha and conidia. Half roots of each banana seedling (30 seedlings) were randomly cut off, 20 seedlings were soaked in pure FOC4 suspension and 10 were soaked in ddH_2_O for 20 minutes, and re-planted in sterilized pots. Then 100 mL of 3-10 cell suspension and ddH_2_O was respectively drenched into soils of each 10 FOC4-soaked seedlings, while 100 mL of ddH_2_O was also drenched into the soils of the ddH_2_O-soaked seedlings. Symptoms were observed every day and pictures were taken in the 16^th^ day after inoculation. Data were subjected to unpaired two-tailed t-test analysis by Graphpad Prism 8.4.1 (*** *p* < 0.001). The marks in the plot area represent the virulence scores of the inoculated seedlings, and the red lines represent the average virulence scores.

In practice, banana wilt disease caused by *F. oxysporum* f. sp. *cubensis* FOC4 was frequently found to be simultaneously present with banana soft rot disease in banana plantations in China. Given that strain 3-10 could inhibit the hyphal growth of this fungal pathogen (Table 1), we evaluated its efficacy in controlling banana wilt, and found that the addition of strain 3-10 evidently reduced the symptoms of leaf yellowing and root atrophy in banana seedlings (Figure 5).

### 3.6. Bacteriostatic Activities in Solid and Liquid Culture Conditions

**Figure 6:**
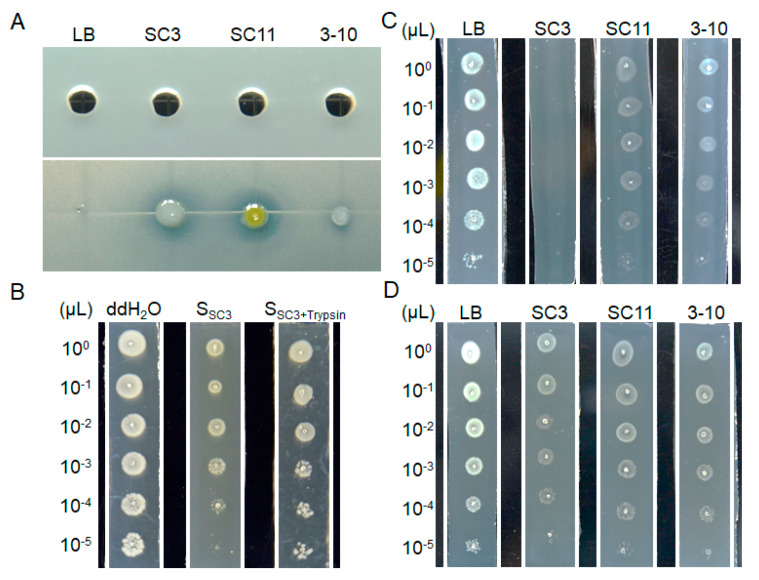
Inhibitory effect of strains SC3, SC11 and 3-10 and their metabolites on the growth of *D. zeae* MS2. (**A**) Inhibitory activities of the SC3, SC11 and 3-10 supernatants (up panel) and 1 µL of overnight cultures (bottom panel) against MS2 growth; (**B**) Inhibitory activity of SC3 supernatant (SSC3) and its Trypsin enzymatic hydrolysate (SSC3 + Trypsin) against MS2 growth; (**C**) MS2 (OD_600_ = 1.5) dilutions respectively grown on the LB agarose, SC3, SC11 and 3-10 metabolite strips; (**D**) MS2 (OD_600_ = 1.5) dilutions respectively grown on the LB agarose, and microwave melted SC3, SC11 and 3-10 metabolite strips.

Toxin bioassays in the above results showed that all the three strains produced diffusible chemical compounds restraining the growth of the tested bacterial pathogens (Figure 1, Table 1). Therefore, we tested the antibiosis activities of the bacterial supernatants against the growth of *D. zeae* MS2, and found it was hardly detectable in 50 μL of supernatants (upper panel in Figure 6A), while obviously visible on the bioassay plate by addition of just 1 μL of bacterial cultures (bottom panel in Figure 6A). A plausible explanation for the seemingly contradictory findings is that the antimicrobial metabolites from the antagonistic bacteria were not abundant by means of liquid culture and completely diffused in the liquid medium, so that there was not a large enough amount of bacteriostatic substances in 50 μL of supernatants to result in detectable antibiosis halos. To verify this explanation, we cultured strain SC3 in liquid LB medium and added the supernatant into the LB agarose medium in a ratio of 7:3, and then tested the growth status of MS2 on the supernatant bioassay plate. The results show that this supernatant plate had certain antibiosis, manifested as the growth status of each dilution on supernatant plate was much worse than that on the control plate, and the 10^-5^ bacterial dilution of MS2 could not survive on the plate (second strip in Figure 6B), whereas it grew well on the control plate (first strip in Figure 6B).

We then tested the antibiosis activities of the three strains by solid culture. Briefly, each of the three overnight antagonistic bacterial cultures was respectively streaked in parallel on LB agarose plates, and the medium containing bacteria was then sliced off, leaving the agar strips containing diffused metabolites. Finally, 1 µL of different dilutions of MS2 overnight culture was progressively spotted onto the metabolite strips. The results show that no dilution of MS2 culture could grow on the SC3 metabolite strip, but all of them survived on the SC11 and 3-10 strips, though they grew much worse than those on the control LB agarose strip (Figure 6C).

### 3.7. The stability of Bacteriostatic Metabolites from the Three Antagonistic Bacteria

Our experiment also showed that after treatment by trypsin for 30 min, the SC3 supernatant dramatically lost the antibiosis activity (third strip in Figure 6B), suggesting that the active compounds of the bacterium are a kind of protein. In addition, after melting the metabolite medium in a microwave oven, parts of the antimicrobial activities of the three strains were lost, especially strain SC3 (Figure 6D), indicating that some of the active antimicrobial compounds produced by the antagonistic bacteria are thermal-sensitive.

## 4. Discussion

In this study, we isolated three strains with strong antagonism to *D. zeae* and identified them as *Pseudomonas fluorescens* SC3, *P. parafulva* SC11 and *Bacillus velezensis* 3-10 by means of MLSA and phylogenetic analyses. In a spot-on-lawn assay, *Pseudomonas* strains SC3 and SC11 formed larger transparent growth inhibition halos against *Dickeya* strains than *Bacillus velezensis* 3-10, whereas smaller halos against other tested pathogenic bacteria in the same cell density were observed (Figure 1A, Table 1), indicating that *Dickeya* strains are more sensitive to the bacteriostatic metabolites produced by *Pseudomonas* strains SC3 and SC11 than those produced by *B. velezensis* strain 3-10.

Our previous study found that both *D. zeae* strains EC1 and MS2 could produce antibiotic-like phytotoxins inhibiting the growth of many pathogenic microorganisms [6,28,45], the former of which has been identified as polyamine zeamines encoded by a *zms* gene cluster [46]. Given that a good biocontrol strain suitable for field application should not be inhibited by the pathogenic target, we tested the susceptibility of the three antagonistic bacteria strains to the metabolites of *D. zeae*. The results show that *D. zeae* rice strain EC1 obviously inhibited all the three antagonistic bacteria, whereas *D. zeae* banana strain MS2 only had a weak inhibitory activity against strain 3-10 (Gram-positive) (Figure 1B). Even so, the biocontrol effect of antagonistic bacteria was still ideal in a potted trial (Figure 3 and Figure 4), and they had the equivalent bacteriostatic effect on EC1 and MS2, compared with that on the non-toxin produced MS3 (Table 1), suggesting that the actions of the three antagonistic bacteria against *D. zeae* are not strain dependent. The results in this experiment further demonstrate that the phytotoxin produced by strain MS2 is different from the zeamines (produced by strain EC1) that have a broad antimicrobial spectrum against both Gram-positive and Gram-negative bacteria [45]. It has a narrower antimicrobial spectrum and weaker inhibitory activity, consistent with our previous study that only inhibited *Escherichia coli* in the tested Gram-negative bacteria and fungal pathogens [6]. This study expanded a Gram-positive bacterium *B. velezensis* as one of the antimicrobial targets of the novel toxins produced by *D. zeae* banana strain MS2.

*D. zeae* has a wide range of hosts, including 11 natural hosts and 35 artificial hosts, resulting in a severe economic loss in crop yield, especially on maize, rice and banana [6]. In this study, we tested the efficacy of the above three antagonistic strains on controlling the soft rot disease on both monocotyledonous and dicotyledonous hosts. The results indicate that all the three strains, especially strain SC3, were effective in controlling the soft rot disease caused by *D. zeae*. Although these three strains were susceptible to *D. zeae* EC1 (Figure 1B), they had expected efficacy on controlling the rice foot rot disease (Figure 4A). In addition, *B. velezensis* strain 3-10 also had effective biocontrol efficacy against banana wilt disease caused by *Fusarium oxysporum* f. sp. *cubensis* (Figure 5). Given that a mixed infection of banana soft rot and banana wilt occurs frequently in fields in South China, *B. velezensis* 3-10 can be developed as a potential biocontrol agent for practical management of banana diseases.

In the antimicrobial spectrum test of three antagonistic bacterial strains, *Pseudomonas* strains SC3 and SC11 were only antibacterial, and *Bacillus* strain 3-10 inhibited both bacterial and fungal pathogens (Table 1). We further considered the reasons for the differences in the antimicrobial spectrum between *Pseudomonas* SC3, SC11 and *Bacillus* 3-10. One is that they may produce totally different types of antimicrobial metabolite, targeting microorganisms in different ranges, and the other is that the Gram-positive 3-10 bacterium may produce additional antibiotics, rather than that produced by SC3 and SC11, specifically acting on fungi. To give a reasonable explanation, we tried to extract the antimicrobial metabolites of the three strains. However, it was hard to obtain bacteriostatic substances from antagonistic strains in liquid culture (Figure 6A). The active metabolites from the three antagonistic bacteria are thermal- and protease-sensitive (Figure 6B,D), suggesting that the active compounds are a kind of protein. Although both SC3 and SC11 belonged to *Pseudomonas*, they probably produce different types of bacteriocins, manifested as their different ranges of antibacterial spectra, different action intensities towards target bacteria in the same cell density (Table 1), stronger antibiosis of SC3 to *Dickeya* strains (Figure 6C) and more thermal sensitivity of SC3 (Figure 6D).

*Pseudomonas fluorescens* is a usual rhizosphere-inhabiting biocontrol agent producing an impressive variety of secondary metabolites, such as 2,4-diacetylphloroglucinol (DAPG), pyoluteorin (PLT), pyrrolnitrin (PRN), phenazines (PHZ) and hydrogen cyanide (HCN) to inhibit fungal phytopathogens [47,48,49,50,51]. It is also a kind of plant growth promoting rhizobacteria, producing siderophores, which chelate iron and other metals, contributing to disease suppression by conferring a competitive advantage to controlling pathogens because the supply of essential trace minerals in natural habitats is limited [52]. In this study, we found that *P. fluorescens* SC3 inhibited the growth of some tested pathogenic bacteria, suggesting that it probably produces secondary metabolites with specific antibacterial activity. To date, several bacteriocins have been well characterized in *P. fluorescens*, e.g., LlpA produced by strain Pf-5 was able to inhibit many *Pseudomonas* strains [53], and tailocins produced by strain SF4c were active against *X. verlcatoria* Xcv Bv-4a with temperature tolerance and no cytotoxic effects on mammalian cells [54]. In this study, *P. fluorescens* SC3 produced a bacteriocin characteristic of a comparably wide antibacterial spectrum (Table 1), except *Pseudomonas* strains (Figure S1), and protease- and thermal-sensitivity (Figure 6B,D).

Previous studies have shown that *Pseudomonas parafulva* (formerly known as *Pseudomonas fulva*) exhibited antagonistic ability against several bacterial and fungal diseases [55,56,57,58]. In *P. parafulva* CRS01-1, several gene clusters associated to controlling the pathogenic fungus and bacteria were identified, including pyrimidine synthesis, benzoate synthesis gene and so on [59]. In *P. parafulva* PRS09-11288, genome analysis identified a phenazine biosynthetic pathway, which can produce the antibiotic phenazine-1-carboxylic acid (PCA) [60]. In *Pseudomonas* spp., PCA is responsible for the biocontrol ability against *Rhizoctonia solani* [61]. At present, the antibacterial substances and mechanism of this kind of bacteria to control pathogens has not been well studied. In this study, strain SC11 produced antibacterial metabolites unable to inhibit the growth of *Rh. solani* (Figure 6), indicating its different nature from PCA. In our assumption, we consider that the antibacterial metabolites produced by SC11 are different from those produced by SC3, which have stronger antibiosis on *D. dadantii* 3937, *R. solanacearum* EP1, *X. campetris* pv. *campetris* Xcc1, *Pantoea* strains (Table 1) and even on *B. velezensis* 3-10 (Figure S1).

*Bacillus* are usually the dominant microorganisms in soil and in plant micro-ecological systems, and currently, lots of natural isolates with great potency have been screened and applied widely in biocontrol of plant disease [62]. *B. velezensis* is a new type of biocontrol factor with inhibitory ability against a broad spectrum of microbial pathogens and plant growth promoting activity [63]. It produces various antifungal and antibacterial metabolites including β-1,3-1,4-glucanase, lipopeptides (iturin, fengycin and surfactin), polyketides (macrolactin, bacillaene and difficidin or oxydifficidin) and peptides (plantazolicin, amylocyclicin and bacilysin) [64,65,66,67]. It is reported that lipopeptides produced by *B. velezensis* are the major contributor to inhibiting pathogenic fungi, such as *F. oxysporum*, *C. gloeosporioides* and so on [27,68]. Recent studies have shown that difficidin and bacilysin produced by *B. velezensis* FZB42 (formerly named *B. amyloliquefaciens* subsp. *plantarum* FZB42) are antimicrobial compounds against *Xanthomonas* strains [67,69]. Our study identified a *B. velezensis* strain 3-10 with a wide antagonistic spectrum of bacterial and fungal pathogens. In the greenhouse trials, strain 3-10 had effective biocontrol efficacy both on banana soft rot and wilt diseases, suggesting that it probably produces a variety of antimicrobial compounds. Further investigation is needed to identify the specific compounds responsible for *D. zeae* growth inhibition.

As is well known, bacteriocins are a kind of bactericidal protein or polypeptide synthesized by ribosomes in bacteria which are immune to the secreted bacteriocin. Early studies suggested that bacteriocins only work on the same or closely related species [54,70,71]. However, in recent years, more and more research results show that some bacteriocins may also have a killing action on other kinds of bacteria. In this study, we found that *P. fluorescens* strain SC3 produced bacteriocin-like metabolites with inhibitory activity against *Dickeya* spp., *X. campetris* pv. *campetris* Xcc1 and *R. solanacearum* EP1, but not against *Pantoea* spp. (Table 1), wider than the action spectrum of LlpA [54]. We also found that the action spectra of the antibacterial metabolites are not relative to the genetic evolution relationship of the antagonistic strains (Figure S3), e.g., SC11 is tolerant to the SC3 metabolites and vice versa (Figure S1), though they both belong to *Pseudomonas*, and the closely related *Dickeya* and *Pantoea* strains showed completely reverse performance on SC3 metabolites-resistance (Table 1), and the effect of SC11 on 3-10 was significantly stronger than that of SC3 (Figure S1).

The three antagonistic bacteria screened in this study display good potency in the biocontrol of the soft rot disease caused by *D. zeae*, especially strain SC3. However, the stability of biocontrol effect is difficult to guarantee, since strains may be sensitive to environmental conditions in the field, like soil and weather conditions [72]. Additionally, application by mixing these three strains seems unfeasible, since strain 3-10 could restrain the growth of the two *Pseudomonas* strains, and vice versa (Figure S1). Therefore, we hope to extract and purify the antibacterial substances of these antagonistic strains in further study, so that they can be applied in agricultural production more effectively and stably.

## 5. Conclusion

In this study, we isolated three antagonistic bacteria from rice and ginger rhizosphere with strong inhibitory effect on *D. zeae* and identified them as *Pseudomonas fluorescens* SC3, *P. parafulva* SC11 and *Bacillus velezensis* 3-10 by means of MLSA and phylogenetic analyses. *Pseudomonas* strains of SC3 and SC11 had an antibacterial spectrum and the *Bacillus* strain 3-10 had a broad antimicrobial spectrum against both the tested pathogenic bacteria and fungi. We further found that all three strains had effective biocontrol efficacy against soft rot disease under greenhouse conditions and *P. fluorescens* strain SC3 had the best performance.

## Figures and Tables

**Table 1:** Antimicrobial distances of antagonistic bacterial strains against some pathogenic microorganisms.

Microorganism	Host	Source or Reference	Antimicrobial Distance (mm)			Inhibition Rate ^a^ of 3–10 (%)
			SC3	SC11	3-10	
*Dickeya zeae* EC1	Rice	[28]	16.64 ± 0.59	7.94 ± 0.49	9.86 ± 0.50	/
*D. zeae* MS2	Banana	[6]	15.20 ± 0.23	8.89 ± 0.29	5.72 ± 0.24	/
*D. zeae* MS3	Banana	[6]	16.48 ± 0.19	9.07 ± 0.13	6.59 ± 0.18	/
*D. fangzhongdai* HK1	Orchid	Lab storage	12.59 ± 0.52	7.87 ± 0.34	3.76 ± 0.23	/
*D. dadantii* 3937	Potato	Lab storage	3.66 ± 0.08	6.64 ± 0.21	4.24 ± 0.18	/
*Ralstonia solanacearum* EP1	Eggplant	Lab storage	3.58 ± 0.14	7.44 ± 0.25	10.26 ± 0.27	/
*Xanthomonas campetris* pv. *campetris* Xcc1	Crucifer	[38]	4.54 ± 0.17	0.86 ± 0.09	11.34 ± 0.83	/
*Pantoea anthophila* CL1	Wampee	[39]	0	3.29 ± 0.19	4.23 ± 0.11	/
*Pa. ananatis* PP1	Peach	Lab storage	0	1.12 ± 0.09	4.58 ± 0.18	/
*Pa. ananatis* SC7	Rice	Lab storage	0	0	5.28 ± 0.10	/
*Colletotrichum capsici*	Capsicum	Lab storage	0	0	9.34 ± 0.20	48.50 ± 1.04
*C. gloeosporioides*	Mango	Lab storage	0	0	8.45 ± 0.23	42.10 ± 1.15
*Fusarium oxysporum* f. sp. *cubensis* FOC4	Banana	[40]	0	0	7.30 ± 0.20	35.06 ± 0.99
*Rhizoctonia solani* AG-1 1A	Rice	[41]	0	0	3.64 ± 0.11	8.68 ± 0.28
*Magnaporthe oryzae* B157	Rice	[42]	0	0	12.27 ± 0.23	80.18 ± 1.49
*Sporisorium scitamineum*	Sugarcane	[36]	0	0	17.04 ± 0.55	34.54 ± 1.11

^a^ The inhibition rate was calculated as: the distance between the antagonistic bacteria and the fungal hyphal edge/the radius of untreated fungal colony × 100%.

## Supplementary Materials

Supplementary materials can be found at https://www.mdpi.com/2076-2607/8/5/697/s1, Figure S1: Inhibitory activities of strains SC3, SC11 and 3-10 against the growth of strains SC3 and SC11 in spot-on-lawn assay, Figure S2: Inhibitory activities of strains SC3, SC11 and 3-10 against the sexual mating of Sporisorium scitamineum, Figure S3: Phylogenetic tree based on the concatenated nucleotide sequences of the 16S rRNA gene of bacterial strains used in this study.
