# Supplementary material for: Screening, Identification and Efficacy Evaluation of Antagonistic Bacteria for Biocontrol of Soft Rot Disease Caused by Dickeya zeae
Source: Microorganisms. 2020 May 9;8(5):697. doi: 10.3390/microorganisms8050697 (PMC7285164; doi:10.3390/microorganisms8050697)
Supplement: Supplementary file 1 [file microorganisms-08-00697-s001.pdf]

Supplemental material

Screening, Identification and Efficacy Evaluation of Antagonistic Bacteria for Biocontrol for Soft Rot Disease Caused by *Dickeya zeae*

Jieling Li[1,†], Ming Hu[1,†], Yang Xue[1], Xia Chen[1], Guangtao Lu[2], Lianhui Zhang[1], Jianuan Zhou[1,*]

[1] Guangdong Laboratory for Lingnan Modern Agriculture, Guangdong Province Key Laboratory of Microbial Signals and Disease Control, Integrative Microbiology Research Centre, South China Agricultural University, Guangzhou 510642, China

[2] State Key Laboratory for Conservation and Utilization of Subtropical Agro-bioresources, College of Life Science and Technology, Guangxi University, Nanning 530004, China

* Correspondence: jianuanzhou@scau.edu.cn

[†] These authors contributed equally to this work

Content:

Figure S1.

Figure S2

Figure S3

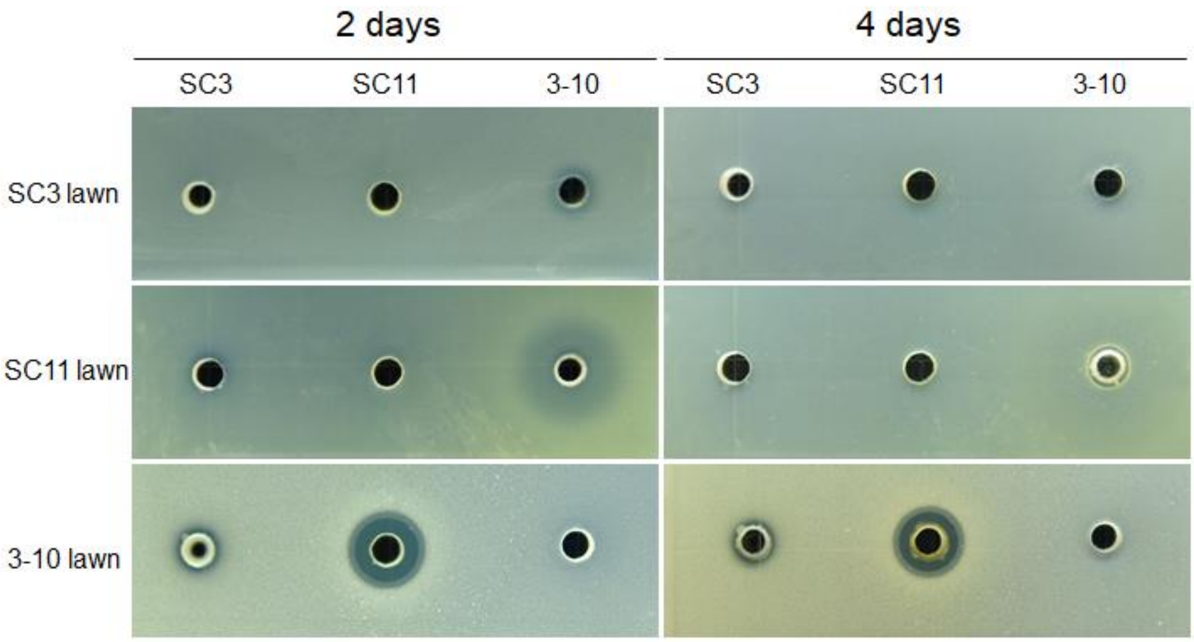

Figure 1. Inhibitory activities of strains SC3, SC11 and 3-10 against the growth of strains SC3 and SC11 in spot-on-lawn assay.

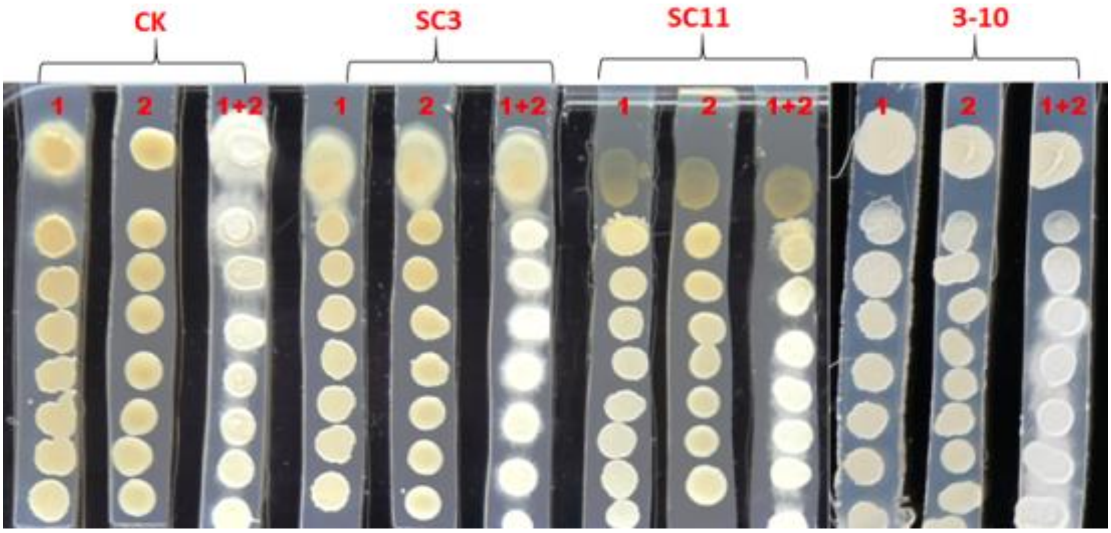

Figure S2. Inhibitory activities of strains SC3, SC11 and 3-10 against the sexual mating of *Sporisorium scitamineum*. PDA plate was cut into separated slices (0.6 cm in width). An aliquot of 1 μL overnight bacterial culture was added on one end of the agar slice, and then the mixture of *S. scitamineum* haploid cells MAT1 and MAT2 was spotted (0.5 μL of $OD_{600} \approx 1.5$) on the slice at progressively further distances from the loaded sample. LB medium was added in the same way in place of bacterial culture as a negative control. The plates were incubated at 28 ^{o}C for 2 days, until the white hypha in the negative control grew to reach the edges of the slice. 1, MAT1; 2, MAT2; 1+2, mixture of haploid cells MAT1 and MAT2.

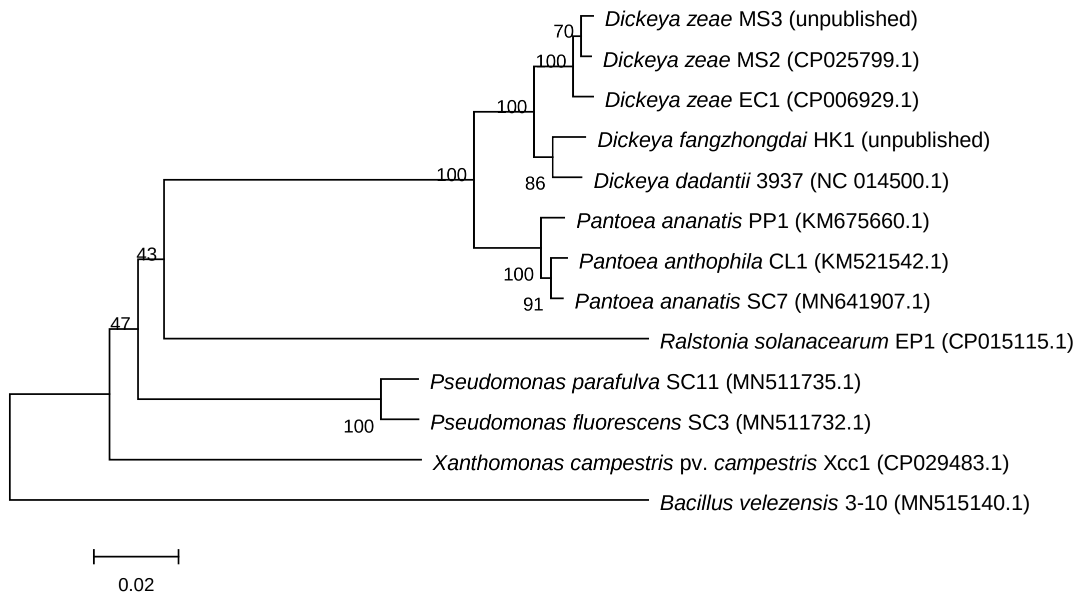

Figure S3. Phylogenetic tree based on the concatenated nucleotide sequences of the 16S rRNA gene of bacterial strains used in this study. Consensus sequences of the 16S rRNA gene were aligned with ClustalW and trimmed in the same size and assembled to construct a Neighbor-joining tree. Bootstrap values after 1000 replicates are expressed as percentages.
